# Caries of Jaw from Impacted Wisdom Tooth

**Published:** 1896-01

**Authors:** R. E. Sparks

**Affiliations:** Kingston, Ont.


ARTICLE VII.

### CARIES OF JAW FROM IMPACTED WISDOM TOOTH.

BY R. E. SPARKS, KINGSTON, ONT.

E. L.——, aged about 27, was admitted to Kingston General Hospital, June 12th, 1895. He had a large swelling at the angle of the lower jaw, left side. I was asked by the attending surgeon to see the case. We found a slight discharge from a small opening opposite the angle of the jaw. He could not open the mouth more than a quarter of an inch at the incisors. With a distender we forced the mouth open to about half an inch. We found teeth good, but wisdom tooth impacted. The history of the case as he gave it to us was, that about March 1st he felt gnawing pains in the region of the angle of the jaw. These became more intense, passing over the side of the head. About the middle of April the jaw swelled, and by May 1st was locked. Consulted a dentist, who diagnosed an impacted wisdom tooth, but said he could not extract it until the swelling disappeared. He visited a doctor, who recommended poulticing. This was done for a week, when the swelling "broke" and the muscles relaxed, allowing him to open his mouth pretty freely. Exposure to cold brought on a relapse. Jaw again became swollen and locked. He again consulted his doctor, who at once sent him to the hospital.

On June 14th an anæsthetic was administered and an exploratory incision was made from the angle of the jaw forward almost to the facial artery. It was found that caries had attacked the jaw opposite the wisdom tooth. This was thoroughly scraped and washed out. The mouth was pried open and the second and third molars removed; the second merely to admit of the removal of the third. It was found that a channel existed from the socket of the wisdom tooth to the external opening just made. The wound was stitched, leaving a drainage tube in. He was dismissed on July 4th. We saw him on August 31st. Swelling entirely disappeared; mouth opened quite freely scar very slightly noticeable. Altogether a very satisfactory result.—*Dominion Dental Journal.*

